# *JACEP Open* Annual Report 2025

**DOI:** 10.1016/j.acepjo.2026.100488

**Published:** 2026-08-19

**Authors:** Marianne Gausche-Hill, Christian Tomaszewski, Juan March, Remle Crowe, Lara N. Goldstein, Mike Wells

**Affiliations:** 1 Departments of Emergency Medicine and Pediatrics, Harbor-University of California Los Angeles Medical Center, Los Angeles, California, USA; 2 Clinical Emergency Medicine, University of California, San Diego, San Diego, California, USA; 3 Division of EMS, Department of Emergency Medicine, East Carolina University, Greenville, North Carolina, USA; 4 Department of Emergency Medicine, University of Texas, San Antonio, Texas, USA; 5 ESO, Inc, Austin, Texas, USA; 6 Department of Emergency Medicine, Memorial Regional Hospital, Hollywood, Florida, USA; 7 Division of Emergency Medicine, Florida Atlantic University, Boca Raton, Florida, USA

**Keywords:** emergency medicine, editor annual report, acceptance rates, production timeline, journal performance, publishing statistics

## 1 Overview

In this report, we summarize the performance and accomplishments of the *Journal of the American College of Emergency Physicians Open* (*JACEP Open)* for 2025. The journal is beginning its seventh year of publication. Beginning in January 2025, Elsevier assumed duties as the publisher of *JACEP Open*.

## 2 Status of the Journal

*JACEP Open* began accepting and publishing manuscripts in the latter half of 2019. Important milestones include listing in PubMed Central, indexing in PubMed, acceptance to the Clarivate Web of Science for coverage in the Emerging Sources Citation Index, indexing with the Directory of Open Access Journals, PubMed, Europe PubMed, Google Scholar, Embase (Elsevier, Inc.), SCOPUS, and SHERPA/RoMEO. The journal’s impact factor for 2025 was 1.9 with a cite score of 2.8. In 2023, the journal launched a special collection in critical care, and in 2024, the journal added special collections in Informatics and Observation Medicine.

## 3 Editorial and Production Performance

The journal received almost 650 manuscripts in 2025 (Table 1), a 13% decrease over the previous year. Original research, brief reports, reviews, and clinical concepts articles comprised approximately 45% of all submissions. Case reports and Images and Videos in Emergency Medicine comprised the remaining 55% of submissions. Approximately 33% of submissions were from international authors from 49 countries outside the United States, and 428 (57%) manuscripts were transferred from *JACEP Open*’s companion journal, *Annals of Emergency Medicine*.

**Table 1:** *JACEP Open* submissions received by article category, by year of publication.

Article type	2020	2021	2022	2023	2024	2025
Abstracts				1	1	1
Brief Commentary	13	5	4	5	1	0
Brief Report	68	47	45	42	63	63
Case Report	134	135	102	107	174	123
Clinical Concepts	69	33	15	20	11	18
Editorial	6	5	4	2	3	2
Images in Emergency Medicine	146	181	124	152	182	157
*JACEP Open* Podcast Summary	3	5	0	0	0	0
Letter to the Editor	14	9	4	3	11	5
Original Research	226	211	153	233	239	229
Review Article	49	20	17	20	18	16
Special Contribution	8	12	9	9	15	12
Systematic Review–Meta-analysis	14	17	16	14	13	13
Videos in Emergency Medicine	0	3	10	17	10	9
Total	750	683	503	625	741	654

The journal accepted 287 articles for 2025. The journal acceptance rate was 36% for original research, brief reports, systematic reviews, review articles, and clinical concepts papers, and these 109 manuscripts spanned a range of topic areas (Table 2). Topic areas were expanded to address the growing expansion of subspecialties in emergency medicine, including Addiction Medicine and Healthcare Administration, Leadership and Management. (Table 2). Accepted manuscripts included authors from 20 different countries.

**Table 2:** Topic areas of original research, brief reports, systematic reviews, reviews, and clinical concepts papers accepted in 2025.

Topic area	No. of articles
Addiction Medicine	1
Airway	1
Cardiology	6
Diversity, Equity and Inclusion	1
Education	1
Emergency Medical Services	12
Evidence-Based Emergency Medicine	1
General Medicine	10
Geriatrics	5
Health Policy	7
Imaging	2
Infectious Disease	7
Informatics	2
Injury Prevention	1
Obstetrics and Gynecology	2
Pain Management and Sedation	3
Pediatrics	11
Physician Wellness	3
The Practice of Emergency Medicine	15
Pulmonary	4
Toxicology	4
Trauma and Injury Prevention	5
Health Care Administration Leadership and Management	1
Total	109

*JACEP Open*’s editorial and production processes continue to meet rapid internal benchmarks, with the median time to first decision submission 24 days. As an open access journal, *JACEP Open* aims to continue to provide a rigorous, robust, and rapid scientific review of manuscripts (Fig).

**Figure:**
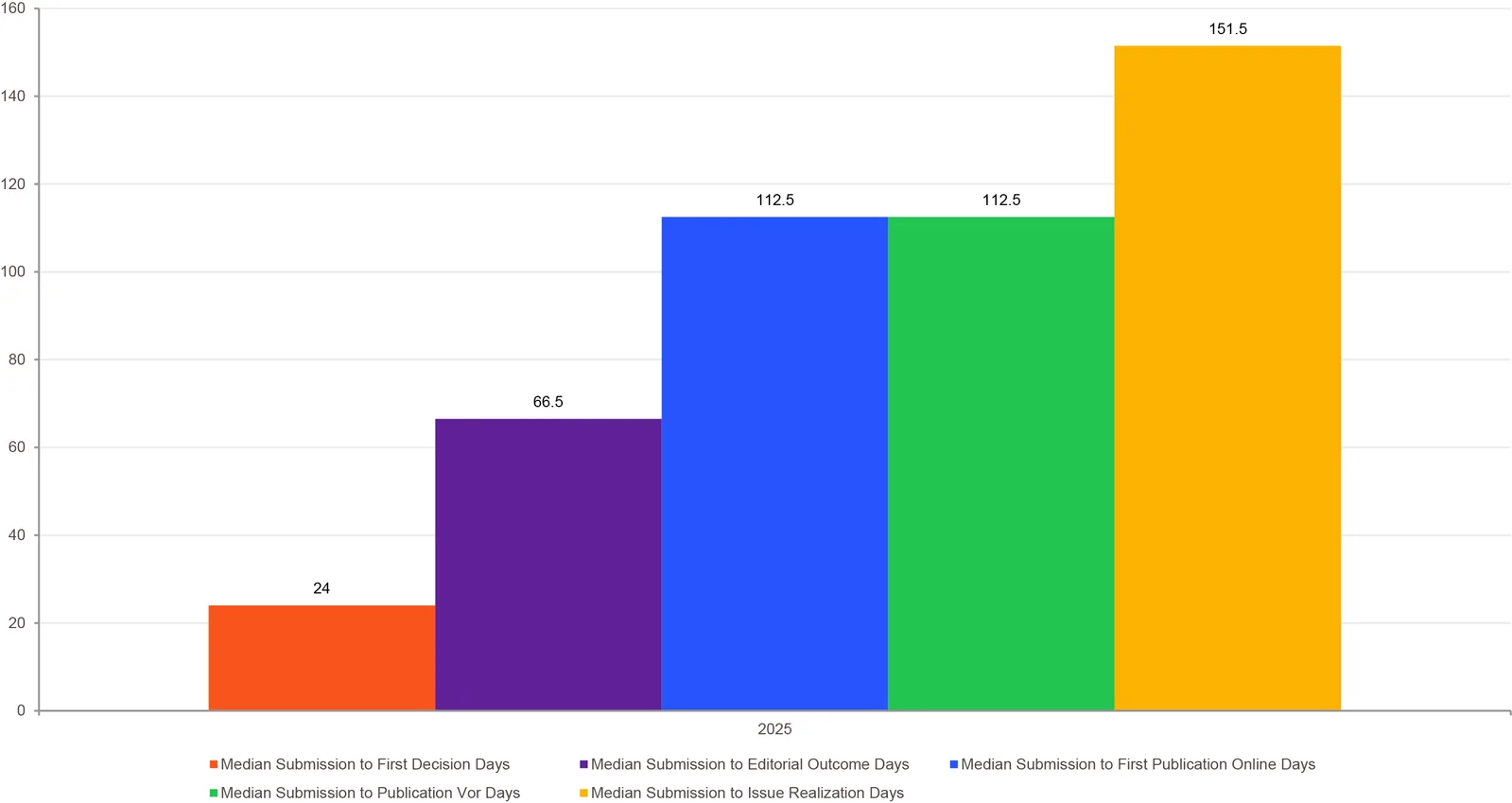
*JACEP Open* median editorial and publication speed for published articles in 2025.

## 4 Manuscript Access and Citations

As an open access journal, all *JACEP Open* content is fully accessible without a subscription fee. Total combined downloads from ScienceDirect, JBS, and Clinical Key were 672,367 in 2025. Table 3 summarizes the journal’s top downloaded articles published in 2025 from jacepopen.com. The countries with the highest number of accepted articles in 2025 were Taiwan, Japan, and China. The journal’s most highly cited articles of all time 2025 are listed in Table 4.

**Table 3:** Top JBS downloaded articles in 2025, published in 2025.

Article title	Corresponding author	Publication date	Article type	JBS usage	Lifetime JBS usage	Lifetime scopus citations
Contemporary Approach to Acute Pancreatitis in Emergency Medicine	Hawatian, Kegham	2025-02-18	Review article	7,410	7,410	0
Sepsis Resuscitation: Time to Embrace a Restrictive Fluid Strategy?	Rodriguez-Guevara, Ignacio	2025-01-21	Correspondence	3,845	3,845	1
Artificial Intelligence in Emergency Medicine: A Primer for the Non-Expert	Smith, Moira	2025-02-14	Full length article	2,522	2,522	2
Burnout, depression and stress in emergency department nurses and physicians and the impact on private and work life: A systematic review	Klingberg, Karsten	2025-02-14	Review article	1,810	1,810	4
Lack of 24/7 Attending Physician Coverage in U.S. Emergency Departments, 2022	Camargo, Carlos A.	2025-02-05	Full length article	1,683	1,683	1
Prehospital Blood Administration in Traumatic Hemorrhagic Shock∗	McNeilly, Bryan	2025-01-24	Full length article	1,649	1,649	1
Extravasation Injuries	Ma, Jinling	2025-01-31	Short Review	1,391	1,391	0
A Case of Intussusception with Bowel Obstruction in a Gastric Roux-En-Y Patient Prescribed Semaglutide	Pavuluri, Suresh	2025-01-30	Case Report	1,372	1,372	0
Preventing unrecognized esophageal intubation in the emergency department		2023-06-01	Full length article	1,365	1,365	5
Emergency Department Placed Central Lines for Trauma Patients: A Retrospective Case Control Study on CLABSI Risk from Central Lines placed Emergently in the ED	Epstein, Larissa	2025-02-13	Full length article	1,343	1,343	0

JBS, jacepopen.com.

**Table 4:** Top cited articles 2025, published all time (Scopus) in *JACEP Open*.

Article title	Corresponding author	Publication date	Article type	Scopus citations	Lifetime SD usage	Lifetime scopus citations
Improving the management of acutely agitated patients in the emergency department through implementation of Project BETA (Best Practices in the Evaluation and Treatment of Agitation)	Lynn P. Roppolo MD	2020-10-01	Review article	19	2862	78
Crucial considerations: Sex differences in the epidemiology, diagnosis, treatment, and outcomes of acute pulmonary embolism in non-pregnant adult patients	Angela F Jarman	2021-02-01	Review article	17	333	44
Emergency medical services targeting opioid user disorder: An exploration of current out-of-hospital post-overdose interventions	James R. Langabeer	2020-12-01	Review article	16	429	35
Implementation of a prehospital whole blood program: Lessons learned	Michael J. Levy	2024-04-01	Full length article	15	414	17
Artificial intelligence in emergency medicine: A scoping review	Sameer Massood	2020-12-01	Full length article	15	1642	66
Hospitals as disaster victims: Lessons not learned?	Chadd Kraus	2022-02-01	Review article	15	834	27
Emergency department visits among people with cancer: Frequency, symptoms, and characteristics	Michael Gallaway	2021-06-01	Full length article	14	642	48
An inventory of stroke centers in the United States	Carlos A. Camargo, Jr	2022-04-01	Full length article	14	203	34
The agitated older adult in the emergency department: a narrative review of common causes and management strategies	Maura Kennedy	2020-10-01	Review article	13	1797	33
From concept to reality: A comprehensive exploration into the development and evolution of a virtual emergency department	Jason Talevski	2024-08-01	Full length article	13	1048	15

## 5 Social Media and Online Presence

*JACEP Open* promotes the journal through social media outlets, including Twitter (X), Facebook, Instagram, Threads, LinkedIn, and most recently on BlueSky. The journal has 807 followers on Twitter and received 29,256 impressions in 2025. Across other platforms, the journal has 515 followers and 639 impressions on Facebook and 275 followers and 5254 impressions on LinkedIn. The Instagram and Threads, which have become a focus in early 2026, currently have 230 and 36 followers, respectively, with continued growth expected with the accounts being used to promote the journal’s Videos of Emergency Medicine articles. There were 539 *JACEP Open* media hits in national, local, and online outlets. Some of the top outlets included the following: Associated Press, Becker's Hospital Review, Boston Herald, Business Insider, Business Wire, BuzzFeed, Drugs.com, EMS Insider, Forbes, Hartford Courant, Healio, HealthLeaders, Houston Chronicle, Markets Insider, Marketwatch, MedPage Today, Medscape, MSN News, News Break, Newsweek, Physicians Weekly, Popular Science, Springer Link, Star-Telegram, The Independent (US), The Mercury News, The Sacramento Bee, US News & World Report, Yahoo! Finance, Yahoo! Lifestyle, Yahoo! News. One of the top hits was for “Economic Benefits to Healthcare Systems with AI-Guided TTE-First Strategies.”

## 6 Editorial Board

The *JACEP Open* editorial board plays a pivotal role in the success of the journal. The current editorial board includes 49 editors, of whom 16 are women, and 2 are international editors. In 2025, we recruited 6 new editors, including 2 new assistant editors for the pediatric section. We also recruited one new assistant methodology editor as part of our ongoing initiative to bolster the journal’s biostatistical expertise. Five individuals serve as methodology statistical editors, assuring expert assessment of all original research manuscripts with more advanced statistical and analytic methods.

## 7 Peer Reviewers

Currently, there are over 561 individuals in *JACEP Open*’s pool of primary reviewers. In 2025, *JACEP Open* peer reviewers completed 556 assignments. *JACEP Open* recognized the top 25 peer reviewers for 2024-2025 (Table 5) and will do so again in 2025-2026. *JACEP Open* offers continuing medical education credits to all peer reviewers, which are offered by the American College of Emergency Physicians and sent at the end of the calendar year.

**Table 5:** Top 25 peer reviewers for 2024 to 2025.

Björn af Ugglas, MSc
FitzGerald Alcindor, MD
Eileen Baker, MD, PhD
Janice C. Blanchard, MD
Erik Blutinger, MD, MSc
Alexander Bracey, MD
John Bruno, MD
Stephen V. Cantrill, MD
James Chan, MD
Esther H. Chen, MD
Dennis Durbin, MD, MSCE
Jason S. Haukoos, MD, MSc
Joshua Krieger, MD
Douglas F. Kupas, MD
John Austin Lee, MD, MPH
Samuel D. Luber, MD, MPH
Juan Carlos Montoy, MD, PhD
Michael Moss, MD
Christopher R. Peabody, MD, MPH
Evan S. Schwarz, MD
Fred Serveryn, MD
Samuel J. Stratton, MD, MPH
Alexander Ulintz, MD
Howard A. Werman, MD
Justin Williams, MD

## 8 Special Collections

In 2023, the journal launched a new special collection in critical care and built upon *JACEP Open*’s existing body of published critical care papers. Fifteen articles have already been published in the Critical Care Special Collection with Robert Levine, MD, currently serving as lead editor. Other special collections include Informatics (14 articles) with lead editors Adam Landman, MD, MS, R. Andrew Taylor, MD, MHS, and Nathan Hoot, MD, PhD and Observation Medicine (7 articles) with lead editors Christopher Baugh, MD, MBA, Matthew Wheatley, MD, and Joseph Lykins, MD, reviewing and publishing virtual submissions.

*JACEP Open* published abstracts from a scientific meeting. The journal published 18 abstracts from the 2025 Pennsylvania College of Emergency Physicians (PACEP) Scientific Assembly.^1^ The publication of additional abstracts from the 2026 PACEP Scientific Assembly can be found in the May 2026 supplement.

## Funding and Support

By *JACEP Open* policy, all authors are required to disclose any and all commercial, financial, and other relationships in any way related to the subject of this article as per ICMJE conflict of interest guidelines (see www.icmje.org). The authors have stated that no such relationships exist.
